# Evidence-Based Strategies to Promote Long-Term Cardiac Implant Site Health: Review of the Literature

**DOI:** 10.7759/cureus.13027

**Published:** 2021-01-31

**Authors:** Leo Licari, Simona Viola, Claudia Carolla, Sofia Campanella, Giuseppe Salamone

**Affiliations:** 1 Surgical, Oncological and Oral Sciences, University of Palermo, Palermo, ITA; 2 Biological, Chemical and Pharmaceutical Sciences and Technologies, University of Palermo, Palermo, ITA; 3 Surgical, Oncological and Oral Sciences, Policlinico Universitario P. Giaccone, University of Palermo, Palermo, ITA

**Keywords:** cardiac implantable electronic device (cied), infection, prevention, microbiology, surgery

## Abstract

Cardiac implantable electronic devices (CIEDs) are commonly used nowadays. The association between CIED placement and infections is responsible for the high mortality and device explantation rate. Since CIED placement has increased in the past decade, CIED-related complications have risen. In order to reduce the CIED-related complications rate, the prevention of device infection represents the main goal. Over time, many different studies have proven the importance of the measures to prevent CIED-related infections. This review aims to collect the actual recommendations for CIED infection prevention, providing an overview of the main evidence-based strategies.

## Introduction and background

Since the development of advanced hardware, such as transvenous electrodes and downsized generators, the placement of cardiac implantable electronic devices (CIEDs) makes available surgical approaches that have been revealed to be easy to perform and safe. This evidence justifies the rapid rise of CIED procedures performed over the past two decades and the wide spectrum of clinical indications designed for its placement [1-8].

The implantation of CIEDs is not free of complications [9]. Infection represents the most critical complication, and it rates between 1% and 7% of device implantation [9]. Infection often requires device explantation; fatalities occur at a rate between 3% and 19% in patients with device infection. The majority of CIED infections are supported by staphylococcal species (60%-80%) such as S. epidermidis and S. aureus. The American Heart Association (AHA)/Heart Rhythm Society (HRS) recommends antibiotic prophylaxis for infection prevention [6,10-13].

This article aims to investigate the recent trends in surgical techniques for CIED implant site management in order to improve or enhance the long-term health of the implant site and device. The research conducted is a review of the literature.

## Review

We conducted a systematic review. A systematic search was performed in MEDLINE, as well as in Scopus, PubMed Publisher, Google Scholar, and the Cochrane Library, with a search period until August 2020. Searches were adapted to each database and carried out using the specific controlled vocabulary of each database, if available, as well as free-text words.

Two reviewers (L.L. and G.S.) independently evaluated the identified records. All records were screened by title and abstract for eligibility, and the full text of the eligible records was assessed. The English language restriction of the articles was set. Data were assembled in a database set up in Microsoft Office Excel 2016 (Microsoft Corporation, Redmond, WA). The data presented in this review were directly abstracted from the original articles. No statistical analyses were performed.

Surgical site infections (SSIs) are defined by the Centers for Disease Control and Prevention (CDC) as an infection that occurs in the part of the body where the surgery took place and includes superficial, deep, and organ space SSIs [14]. Prevention of SSIs is focused upon three moments in the surgical operation that the World Health Organization (WHO) summarizes in pre, intra, and postoperative measures [15].

Preoperative measures

Patients with fever for 24 hours before implantation are at high risk of CIED infection, and the device placement is recommended to be postponed [16]. Every sign of infection should be screened before CIED implantation and if the infection is confirmed, it should be adequately treated prior to performing the operation [17].

The CDC recommends not to shave the skin perioperatively because it is related to a higher risk of SSIs. Therefore, the CDC recommends cutting the body hair around the incision area only if they interfere with the operation [14].

A preoperative shower or bath with an antiseptic agent is strictly recommended, and it should be done the night before surgery [14]. Finally, antiseptic skin preparation prior to surgery is mandatory for SSI prevention [14].

Even though cardiovascular surgery is a clean surgery, the risk of postoperative SSIs is still present and relatively low. The occurrence of SSI could represent a life-threatening condition. Therefore, systemic prophylactic preoperative antibiotics are routinely used. At present, there are not still guidelines about their use in CIED implantation, but different studies report an advantage in their use [16,18-20]. First-generation cephalosporins, such as cefazolin 2 g, is effective for prophylaxis. It should be administered intravenously (IV) at least 60 minutes before the procedure. If penicillin allergy is known, clindamycin 600 mg IV or vancomycin 1 g IV should be administered 60 minutes prior to the procedure [21].

Finally, the CDC strictly recommends abstaining from smoking for at least one month prior to elective surgery and adequately controlling serum blood glucose levels in diabetic patients. Other host factors associated with a greater risk of CIED infection are often unmodifiable and are represented by comorbidities or drug intake [16]. The risk of infection is also related to the type of device implanted.

Intraoperative measures

The role of the preoperative surgical scrub is well-established [22]. Protocols require that physicians perform surgical scrub, including the hands and the forearms, for at least five minutes with antimicrobial soap or an alcohol-based hand rub. Physicians must therefore dress in a sterile gown and gloves for the duration of the surgical procedure.

The surgical technique must be rigorous and must give attention to sterility throughout the operation. CIEDs are implanted in pockets fashioned in the pre-pectoral subcutaneous space, avoiding fascia incision. Traumatic injury to the small vessels and nerve system can cause accidental bleeding, postoperative pain, and hematoma formation. The dissection of the subcutaneous space must be carefully performed looking for adequate hemostasis. Hematoma must be avoided because it represents a major risk factor for SSI. Irrigation of the pocket prior to definitive suture helps reveal causes of bleeding. It also determines the mechanical debris removal and dilution of contaminants. The effectiveness of prophylactic intrapocket antibiotics in CIEDs has not been proved. The use of monofilament resorbable sutures for closure of the pocket is recommended. The administration of hemostatic agents in the surgical pocket improves postoperative healing reducing the hematoma formation rate. The use of closed incision negative pressure systems (ciNPTs) helps prevent SSIs and CIEDs removal [23].

Postoperative measures

The wound must be maintained clean and dry. If a hematoma occurs, it should be treated conservatively. Hematoma must be evacuated if it increases the skin tension, determining the risk of wound dehiscence. The evacuation of the hematoma must be performed in sterile conditions avoiding contamination of the CIED. The evidence shows that the routine administration of postoperative antibiotics is not useful and the American Heart Association (AHA) guidelines [24] do not recommend their routine use. Postoperative antibiotics should be used only if signs of SSI are present, and they should be administered on culture specimen examination and antibiogram.

Infection of cardiac devices remains a serious problem despite improvements in implantation techniques. Recently, Uslan et al. demonstrated that the estimated CIED infection rate is about 1.9 per 1,000 device-years implanted [10]. The importance of species characterization in SSIs was proven elsewhere [25-33]. The main species identified is the Staphylococcal that accounts for more than 66% of CIED infections. Coagulase-negative Staphylococcus species are a common cause of microbiological specimen contamination. One of the most important virulence factors for some of these pathogens is biofilm formation. The remaining 33% of CIED infections are caused by Gram-negative bacilli and Candida species [34].

The mechanism commonly described for CIED infection is represented by the contamination of the pocket at the time of device implantation, or postoperatively, following a cutaneous infection or the contamination of the device itself or its leads. The infection can spread along the electrode to the endocardium. Another mechanism of infection is the hematogenous seeding of the electrode during bacteremia from a focal infection located elsewhere in the body. Recent studies reported that the estimated endocarditis rate developing by the pocket infection is about 25% of the CIED infections [35].

The signs of CIED infection are represented by an inflammatory reaction or cutaneous erosion. The local signs of infection are often accompanied by pain and fever. Local signs of infection include erythema, fluctuance, wound dehiscence, tenderness, and purulent spillage [34].

When a CIED infection occurs, its management is based on specific antibiotic therapy administration and, eventually, complete removal of the device. Removal of an infected CIED can be performed either by percutaneous lead traction through the generator pocket or by a surgical approach [34]. The use of advanced medications, such as ciNPT and vacuum-assisted closure (VAC) therapy, can be considered to improve healing [23].

The identification of the risk factors for CIED complications is part of the prevention. Risk factors can be divided into patient-related and procedure/device-related. Among the first, gender, age, and comorbidities are recognized as major risk factors. The use of certain medications related to the comorbidities are recognized as risk factors too. Among the second, the lack of rigorous asepsis protocols adoption and postoperative surgical site occurrences (SSOs) are recognized as major risk factors.

## Conclusions

SSI is a serious postoperative complication of CIED implantation that could require device explantation and delay the treatment of cardiac rhythm disease. It sometimes results in death. The best SSI control is its prevention. The most important factors in SSI prevention are rigorous operative technique, antibiotic prophylaxis, and the use of advanced medications such as ciNPTs.
